# Genome-Wide Association Study (GWAS) for Identifying SNPs and Genes Related to Phosphate-Induced Phenotypic Traits in Tomato (*Solanum lycopersicum* L.)

**DOI:** 10.3390/plants13030457

**Published:** 2024-02-05

**Authors:** Haroon Rashid Hakla, Shubham Sharma, Mohammad Urfan, Rushil Mandlik, Surbhi Kumawat, Prakriti Rajput, Bhubneshwari Khajuria, Rehana Chowdhary, Rupesh Deshmukh, Rajib Roychowdhury, Sikander Pal

**Affiliations:** 1 Plant Physiology Laboratory, Department of Botany, University of Jammu, Jammu 180006, India; haroonhakla@rediffmail.com (H.R.H.); shubhamsharma199425@gmail.com (S.S.); urfanbutt1992@gmail.com (M.U.); prakritirajput2512@gmail.com (P.R.); bhubneshwarikhajuria@gmail.com (B.K.); rehanachowdhary2937@gmail.com (R.C.); 2 Central Integrated Pest Management Centre (CIPMC), Srinagar 190008, India; 3 ICAR-National Bureau of Plant Genetic Resources, New Delhi 110012, India; rushilmandlik91@gmail.com (R.M.); surbhikumawat002@gmail.com (S.K.); rupesh0deshmukh@gmail.com (R.D.); 4 Department of Biotechnology, Central University of Haryana, Mahendergarh 123031, India; 5 Department of Ecology and Evolutionary Biology, University of Kansas, Lawrence, KS 66045, USA; 6 Department of Plant Pathology and Weed Research, Institute of Plant Protection, Agricultural Research Organization (ARO)—Volcani Center, Rishon LeZion 7505101, Israel

**Keywords:** tomato, genotype-by-sequencing, gene, GWAS, SNP, phosphate uptake

## Abstract

Phosphate (P) is a crucial macronutrient for normal plant growth and development. The P availability in soils is a limitation factor, and understanding genetic factors playing roles in plant adaptation for improving P uptake is of great biological importance. Genome-wide association studies (GWAS) have become indispensable tools in unraveling the genetic basis of complex traits in various plant species. In this study, a comprehensive GWAS was conducted on diverse tomato (*Solanum lycopersicum* L.) accessions grown under normal and low P conditions for two weeks. Plant traits such as shoot height, primary root length, plant biomass, shoot inorganic content (SiP), and root inorganic content (RiP) were measured. Among several models of GWAS tested, the Bayesian-information and linkage disequilibrium iteratively nested keyway (BLINK) models were used for the identification of single nucleotide polymorphisms (SNPs). Among all the traits analyzed, significantly associated SNPs were recorded for PB, i.e., 1 SNP (SSL4.0CH10_49261145) under control P, SiP, i.e., 1 SNP (SSL4.0CH08_58433186) under control P and 1 SNP (SSL4.0CH08_51271168) under low P and RiP i.e., 2 SNPs (SSL4.0CH04_37267952 and SSL4.0CH09_4609062) under control P and 1 SNP (SSL4.0CH09_3930922) under low P condition. The identified SNPs served as genetic markers pinpointing regions of the tomato genome linked to P-responsive traits. The novel candidate genes associated with the identified SNPs were further analyzed for their protein-protein interactions using STRING. The study provided novel candidate genes, viz. *Solyc10g050370* for PB under control, *Solyc08g062490*, and *Solyc08g062500* for SiP and *Solyc09g010450*, *Solyc09g010460*, *Solyc09g010690*, and *Solyc09g010710* for RiP under low P condition. These findings offer a glimpse into the genetic diversity of tomato accessions’ responses to P uptake, highlighting the potential for tailored breeding programs to develop P-efficient tomato varieties that could adapt to varying soil conditions, making them crucial for sustainable agriculture and addressing global challenges, such as soil depletion and food security.

## 1. Introduction

Tomato (*Solanum lycopersicum* L.) is one of the world’s most commonly used horticultural crops [1,2]. Tomato’s adaptability to diverse environmental conditions and its genetic diversity make it an excellent model for studying plant responses to nutritional stresses [3]. Phosphorus (P), an essential macronutrient, plays a key role in plant growth and development. However, P availability in many agricultural soils is often limited, leading to reduced tomato yield and quality [4]. In India, tomato cultivation is diverse, with a rich repository of traditional and improved accessions representing a wide spectrum of genetic diversity [5]. Understanding how these accessions respond to the variations in P availability is crucial for its sustainable cultivation in the era of climate change and dwindling P resources. Phenotypic traits such as shoot height, primary root length, plant biomass (PB), shoot inorganic phosphate (SiP) content, and root iP content (RiP) provide valuable insights into a plant’s ability to adapt to P-limiting stress conditions. The assessment of these traits under varying P conditions will help to identify accessions with desirable characteristics for P-efficient agricultural practices by requiring lesser P inputs in the crop fields. Additionally, by elucidating the interplay between genetic diversity and P responses, this study can inform future breeding programs to improve tomato productivity and nutrient efficiency [6]. This research bridges the knowledge gap by exploring the intricate relationship between tomato diversity and the P uptake potential of tomato accessions.

The genome-wide association study (GWAS) has become an invaluable tool in genetics and genomics-assisted breeding for unraveling the intricate genetic foundations of multifaceted characteristics found across different crop species such as tomatoes [7]. By employing GWAS, scientists can pinpoint specific variations in single nucleotides (known as single nucleotide polymorphisms or SNPs) that correlate with particular traits of interest, such as plant height or tolerance to stress conditions like drought and salinity in soybean [8]. The application of GWAS in the identification of novel candidate genes for several agronomic traits in important cash crops is widely documented. For instance, GWAS has been used to identify SNPs associated with variations in shoot height, primary root length, PB, SiP, and RiP content in cotton under different regimes of phosphate supply [9]. Similarly, GWAS has been conducted to identify the SNPs and novel candidate genes associated with phenological and agronomic traits in mungbean. Furthermore, higher expression of genes, viz. *VRADI01G08170*, *VRADI11G09170*, *VRADI02G00450*, *VRADI01G00700*, *VRADI07G14240*, *VRADI03G06030*, *VRADI02G14230*, *VRADI08G01540*, *VRADI09G02590*, *VRADI08G00110*, *VRADI02G14240*, and *VRADI02G00430* in the roots, cotyledons, seeds, leaves, meristems, and flowers were linked with traits of phenological and agronomic importance [10]. The key genes associated with fatty acid content were identified in *Brassica napus* using GWAS and significant SNP [11]. In a study involving a panel of 300 inbred rapeseed lines, researchers identified 148 SNPs associated with various traits through mixed linear model (MLM) analysis. Notably, among these SNPs, 30 specific loci situated on the A08 and C03 chromosomes were concurrently linked to three distinct traits: erucic acid, oleic acid, and linoleic acid contents [11]. In the context of tomato, the application of GWAS for the identification of novel candidate genes associated with agronomic traits is widely used, but root traits are the least explored. For instance, the marker-trait association for 28 volatile parameters affecting tomato flavor has been carried out using a mixed linear model (MLM) approach of GWAS on 174 tomato accessions [12]. A meta-analysis of GWAS has successfully provided a list of candidate genes controlling tomato flavor when conducted on 775 tomato accessions. Around 305 noteworthy connections were established between genetic markers and the levels of sugars, acids, amino acids, and volatile compounds associated with flavor [13]. The correlation between vitamin E levels and inherent diversity in chorismate metabolism has been extensively investigated in tomatoes through the utilization of GWAS [14]. Quantitative trait loci (QTL) mapping performed on Multi-parent Advanced Generation Intercross (MAGIC) tomato population under water deficit and salinity stress has revealed specific QTLs linked with plant growth and fruit quality in tomato [15]. Similarly, GWAS has been used to identify the genetic components accountable for reduced salinity tolerance in domesticated lines of tomato. The GWAS conducted on the root Na^+^/K^+^ ratio across 369 different tomato accessions unveiled substantial natural diversity, which notably highlighted the significant role of the *SlHAK20* gene, responsible for encoding HAK/KUP/KT transporters. Moreover, the function of *SlHAK20* was linked to the transportation of both Na^+^ and K^+^ ions, governing their balance and maintaining homeostasis, particularly under salt stress conditions [16]. In tomato, GWAS application for the identification of novel candidate gene (s) associated with natural variation in P uptake and root growth is the least explored so far. In this context, a comprehensive study was conducted on a panel of 103 diverse tomato accessions to analyze root growth variation and root–shoot Pi content. The GWAS performed on tomato composite lines (84 lines) among a panel of 103 diverse accessions under control and low phosphate conditions has provided significant SNPs and genes linked with traits, viz. plant biomass, root, and shoot P contents, were mapped.

## 2. Materials and Methods

### 2.1. Plant Material, Growth Condition, and Treatment

The seeds of 103 diverse tomato accessions were provided by the National Agri-Food Biotechnology Institute, Mohali, India. The experiment was conducted at the botanical garden (32.72° N, 74.85° E) of the Department of Botany, University of Jammu, Jammu, India. The germination sheet roll method of hydroponics was used for growing plants under the control and low phosphate conditions for two weeks. In this method, germination paper was moistened and treated with a broad-spectrum fungicide— (Mancozeb of Dhanuka M-45) and rewashed with double distilled water. A germination sheet was spread, and sterilized seeds were arranged in rows at regular intervals of about 5 cm and were placed in a 2 L bucket containing a half-strength Hoagland solution with a normal (control) phosphate (P) concentration of 40 ppm and low P condition (LP) of 4 ppm respectively. Data were taken from each accession with ten biological replicates (*n* = 10).

### 2.2. Plant Phenotyping

Germination sheet rolls containing young seedlings 14 days after sowing (DAS) in a hydroponic system were harvested. The phenotyping traits including shoot height (SH, cm), primary root length (PRL, cm), and plant dry biomass (g) were measured as stated by Pal et al. [17].

### 2.3. Inorganic Phosphate (iP) Estimation

The inorganic phosphate (iP) content representing a free form of P was determined with the phosphomolybdate method. In brief, 50 mg of fresh plant tissues (root, stem, and leaf) was taken in a falcon tube filled with 3 mL of distilled water and was heated at 80 °C in a water bath for 45 min. A reaction mixture of 5 mL was prepared to consist of 2 mL of solution (1.9 mL deionized water + 100 µL standard solutions/ unknown sample), 1 mL of 0.02 M ammonium molybdate, 1 mL of 0.57 M ascorbic acid, and 1 mL of 3 M sulfuric acid. The solution was incubated at room temperature for 15 to 20 min, such that a blue color developed. The blue phosphomolybdate complex intensity was measured spectrophotometrically at 820 nm. The intensity of the complex formed (blue color) was directly proportional to the amount of iP present in the sample. A known P concentration was used to get a standard calibration curve [18].

### 2.4. Genome-Wide Association Study (GWAS)

In brief, genotype-by-sequencing (GBS) data were obtained for 84 tomato accessions (composite varieties/pure lines, excluding 20 hybrid accessions), from a diverse panel of 103 tomato accessions comprised of 84 composite varieties/pure lines and 20 hybrids. The GBS was performed using 96 plex libraries Hi-seq 10X in the MiSeq System. For performing GWAS and linking P-related traits such as plant biomass (PB), SiP, and RiP of the tomato panel of 84 genotypes, in-house GBS data were procured. The GBS data were arranged and all the quality controls were performed using Trait Analysis by aSSociation, Evolution, and Linkage (TASSEL) version 5.0. The GWAS on various parameters was analyzed using the Genome Association and Prediction Integrated Tool (GAPIT) in the *R* platform. For SNP analysis, a total of 74,384 SNPs were used after a quality control (QC) check using the TASSEL 5.0. Across the panel of genotypes, the SNPs having minor allele frequency (*MAF*) > 0.05 were removed from the analysis. The different models, viz. MLM (mixed linear model), weighted MLM, and enriched compressed MLM (ECMLM) were applied to prevent spurious associations. The Manhattan plot and HapMap were produced by GAPIT version 3 using *R*. For the final analysis for finding significant SNPs, the GWAS used the Bayesian Linear Regression for Mixed Model Association (BLINK) model [19]. It combines traditional linear regression with Bayesian techniques and mixed model approaches to account for population structure and relatedness between individuals [20]. The key advantages of using the BLINK model in the GWAS include its ability to mitigate false positive associations caused by population stratification and relatedness, making it particularly suitable for diverse study populations. The BLINK model also offers improved statistical power to detect true genetic associations, thereby increasing the likelihood of identifying relevant genetic variants responsible for complex traits or diseases [21]. Candidate genes were mapped on the tomato genome browser [https://plants.ensembl.org/Solanum_lycopersicum, accessed on 3 October 2023] within 70–120 kilobases from the identified SNP.

### 2.5. In Silico Gene Expression Analysis

The protein sequence of candidate genes was obtained from UNIprot database [https://www.uniprot.org/uniprotkb, accessed on 3 October 2023] and was used as a query sequence to predict the interacting proteins using the STRING database (https://string-db.org, accessed on 3 October 2023). The gene expression of some candidate genes was analyzed using publicly available transcriptomic datasets [22].

## 3. Results

The present study used a GWAS statistical approach to find the association of the whole genome with the phenotypic traits of tomato accession in response to low P stress, and finally able to map potential candidate genes with in silico validation.

**Figure 1:**
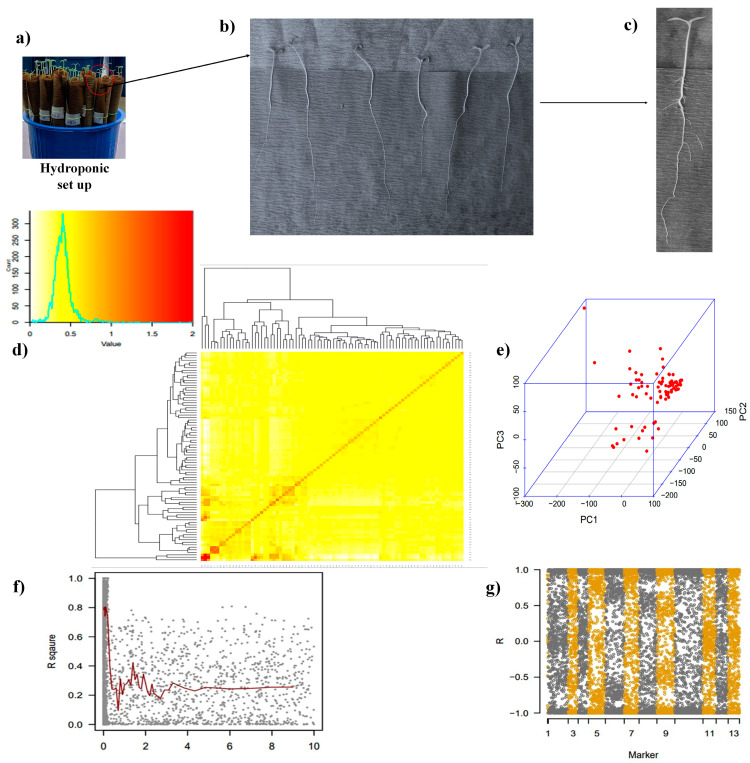
(**a**) Tomato accessions in germination sheet roll under the control (40 ppm) and low phosphate (LP, 4 ppm) conditions, (**b**) tomato accession grown in germination sheet roll at 14 days after sowing (14 DAS), (**c**) tomato young plant shown at 14 DAS, (**d**) kinship matrix of tomato accessions showing closeness among themselves, (**e**) three-dimensional principal component analysis (PCA) illustrated the population structure based on first three PCA components, (**f**) a linkage disequilibrium (LD) plot representing the average genome-wide LD decay in the panel with genome-wide markers in tomato. The *Y*-axis values represent the squared correlation coefficient r^2^, while the *X*-axis represents the physical distance in kilobase (kb), (**g**) heat map representing the marker density in 12 chromosomes.

Tomato accessions grown in germination sheet paper rolls for two weeks under the control and low P conditions showed a huge diversity in terms of phenotypic traits analyzed (Figure 1a–c). Among a collection of 103 accessions, 84 genotypes were sequenced by the genotype-by-sequencing (GBS) method, and a kinship matrix showed the closeness among these panels of accessions (Figure 1d). The three-dimensional principal component analysis (3D-PCA) also provided a significant observation on the population structure of the tomato panel used for the GWAS analysis (Figure 1e). The linkage disequilibrium (LD) plot showed the LD decay, an important attribute for calculating the threshold of the GWAS (Figure 1f). The density of the SNPs over 12 tomato chromosomes also depicts the genetic diversity of the tomato panel used for the GWAS (Figure 1g).

### 3.1. Morphological and Physiological Diversity among Tomato Accessions

**Figure 2:**
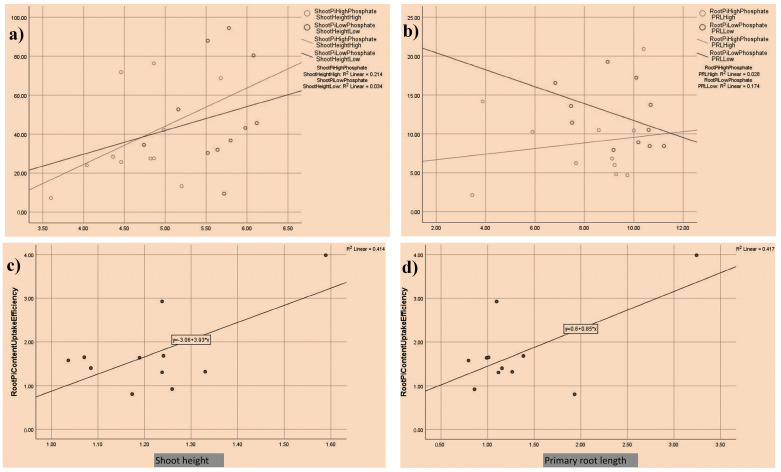
Correlation between traits analyzed. A linear correlation between shoot height and shoot inorganic phosphate content under the control (40 ppm) and low phosphate conditions (LP, 4 ppm) (**a**), primary root length (PRL) and root inorganic phosphate content in high (control) and low phosphate conditions (**b**), root iP content uptake efficiency and shoot height (**c**), and root iP content uptake efficiency and primary root length (PRL) (**d**).

The shoot height (SH, in cm), plant biomass (PB, in g), primary root length (PRL, in cm), SiP, and RiP were measured in a collection of 103 tomato accessions. The maximum SH of 6.88 cm and a minimum of 3.46 cm were noted in AC86 and AC292 in the control conditions. Under low phosphate (LP), maximum (6.94 cm) and minimum SH (2.4 cm) were noted for AC310 and AC223, respectively (Figure S1a). Among a panel of tomato accessions, in the control conditions, the highest PB (0.3041 g) was noted in accession AC156, while the lowest (0.1 g) was in AC17. In low P conditions, the highest PB (0.35 g) and lowest of 0.05 g were noted for AC218 and AC, respectively (Figure S1b). The PRL is an important root trait that is directly associated with P uptake from agricultural soil. In the present study, both under the control and low P conditions, PRL showed a huge diversity. Under the control and low P conditions, maximum and minimum PRL was noted for accessions AC86 (14.34 cm), and AC225 (3.46 cm) under the control conditions, while AC218 (13.54 cm), and AC296 (2.68 cm) under low P, respectively (Figure S1c). Similarly, for control and low P conditions, diversity in terms of SiP and RiP was noted in tomato accessions. The RiP plays an important role in the iP uptake, while SiP indicates shoot iP content available at any given point in time. The highest SiP content was recorded for accessions AC309 and AC36 under the control and low P conditions (Figure S2a). The highest RiP contents were noted for AC310 and AC38, respectively, under the control and low P conditions (Figure S2b). Furthermore, correlation analysis showed that there is a direct linear correlation between several traits measured (Figure 2a–d). For instance, shoot height and shoot iP content under the control P condition showed a stronger linear correlation, with an *R*^2^ value of 0.214 compared to 0.034 in low P conditions (Figure 2a). The correlation between PRL and RiP content was stronger under low P (*R*^2^ of 0.174) compared to the control P condition (*R*^2^ of 0.028) (Figure 2b). No significant change in the linear correlation between shoot height and root iP content uptake efficiency (*R*^2^ of 0.414) was observed (Figure 2c). Similarly, PRL and root iP content uptake efficiency (*R*^2^ of 0.417) showed no significant change in linear correlation (Figure 2d). Furthermore, based on the performance of tomato accessions under the control and LP conditions in terms of SiP, RiP, and root/shoot ratio, they were categorized into tolerant and sensitive accessions [23,24] (Figure S3a–c).

### 3.2. GWAS Analysis

The GWAS analysis was performed on this panel of 84 accessions subjected to the control and low P conditions. The BLINK model of the GWAS was performed with the GAPIT tool using *R*-studio version 4.2.2 for all the morphological (shoot height, primary root length, and plant biomass) and physiological (shoot inorganic phosphate and root inorganic phosphate) traits measured. In the control P condition, significant SNPs were detected in PB, SiP, and RiP, whereas in the case of the low P condition, significant SNPs were identified in SiP and RiP.

**Figure 3:**
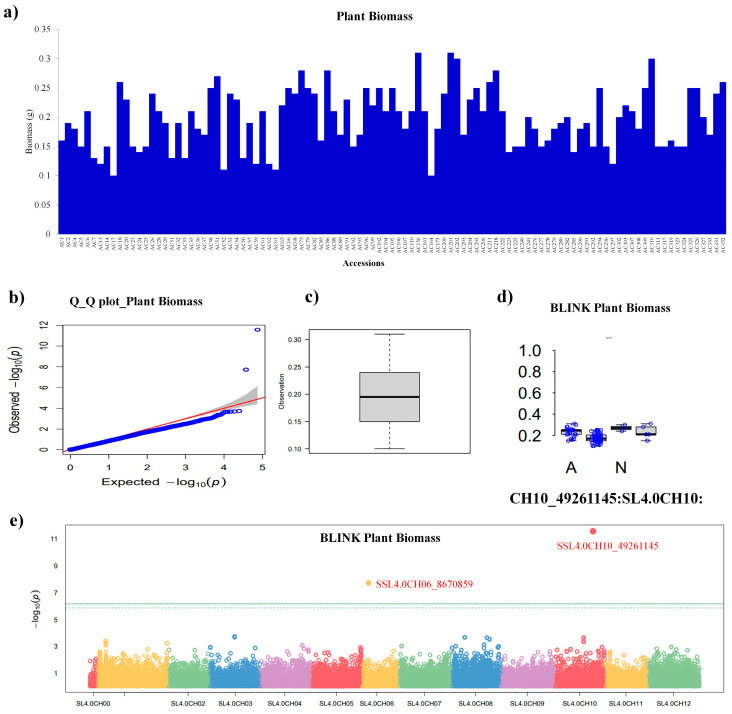
Plant Biomass and GWAS. (**a**) Plant biomass (in g) of tomato accessions under the control, (**b**) Manhattan plot showing significant SNPs, viz. SSL4.0CH10_49261145 and SSL4.0CH06_8670859, is located on chromosome numbers 10 and 6, while the green horizontal line indicates the significance threshold, (**c**) quantile–quantile (Q-Q) plots based on observed versus expected −log_10_ (*p*-values) of plant biomass, (**d**) whisker plot showing significant SNPs pattern (**e**) showing significant SNPs distribution pattern on chromosome number 6 and 10. Different colors in (**e**) indicate different chromosomes.

Plant biomass (PB) under the control showed diversity in the tomato panel (Figure 3a). The Manhattan plot showed that two significant SNPs, viz. SNP SSL4.0CH10_49261145 and SSL4.0CH06_8670859 were located on chromosomes 10 and 6 above the threshold line (Figure 3b). The Q-Q plot showed the behavior of the PB trait in terms of expected and observed log values (Figure 3c). Similarly, the whisker plot showed the behavior of SNPs identified for PB trait among a panel of tomato accessions in the control condition (Figure 3d). The distribution of significant SNP on the chromosomes is shown in Figure 3e. The tomato panel under low P conditions (LP) analyzed using the GWAS procedure showed no significant SNPs above the threshold line, so no further analysis was performed.

**Figure 4:**
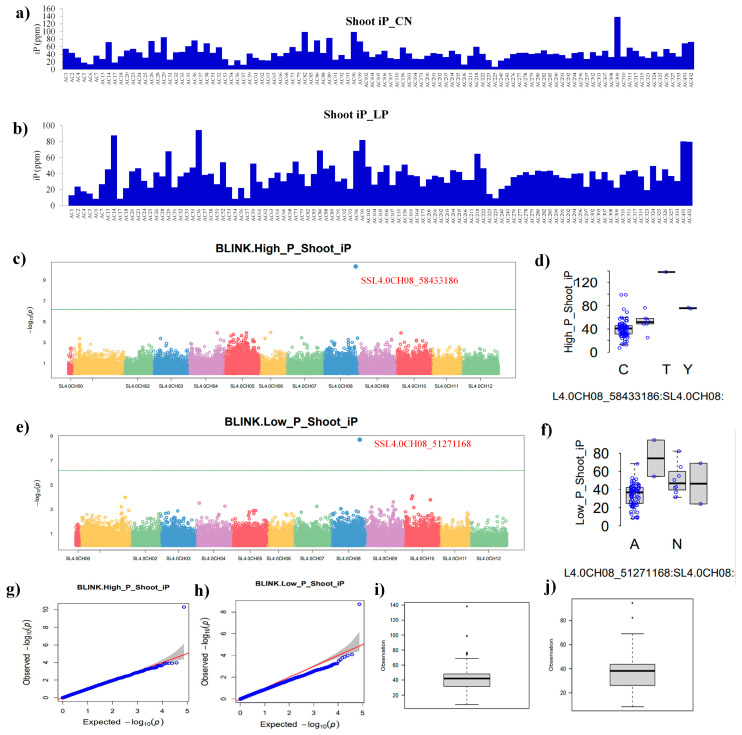
Shoot iP and GWAS. Shoot inorganic phosphate content (SiP, ppm/mg fresh weight) of tomato accessions under control (**a**) and low phosphate treatment (**b**), Manhattan plots showing significant SNPs, viz. SSL4.0CH08_58433186 located on chromosome number 8 in control (**c**), and significant SNPs distribution pattern on chromosome number 8 (**d**), Manhattan plot showing SNP SSL4.0CH08_51271168 on chromosome 8 under low phosphate condition (**e**), and significant SNPs distribution pattern on chromosome number 8 (**f**), the green horizontal line indicates the significance threshold. The quantile–quantile (Q-Q) plots based on observed versus expected −log_10_ (*p*-values) and whisker plots show the behavior of the SiP in control (**g**,**h**) and low phosphate condition (**i**,**j**).

For shoot inorganic phosphate (SiP), variation in the tomato panel was observed under the control and LP conditions (Figure 4a,b). The Manhattan plot of SiP under the control conditions showed a single SNP above the threshold line (Figure 4c), while the distribution of significant SNPs on chromosome 8 is provided (Figure 4d). The BLINK model of the GWAS provided one significant SNP SSL4.0CH08_58433186 localized on chromosome 8. The Q-Q and whisker plots provided information about the behavior of the SiP trait in the tomato panel under the control conditions (Figure 4g–i). For SiP under the LP conditions, the tomato panel showed huge diversity in terms of the iP content (Figure 4b). The BLINK provided a single SNP, viz. SSL4.0CH08_51271168, localized on chromosome 8. The Manhattan plot showed a single SNP above the threshold line (Figure 4e). The distribution pattern of significant SNPs under the LP conditions on chromosome 8 is shown in Figure 4f. The Q-Q and whisker plot showed the behavior of the SiP trait under the LP conditions in the tomato panel (Figure 4h–j).

**Figure 5:**
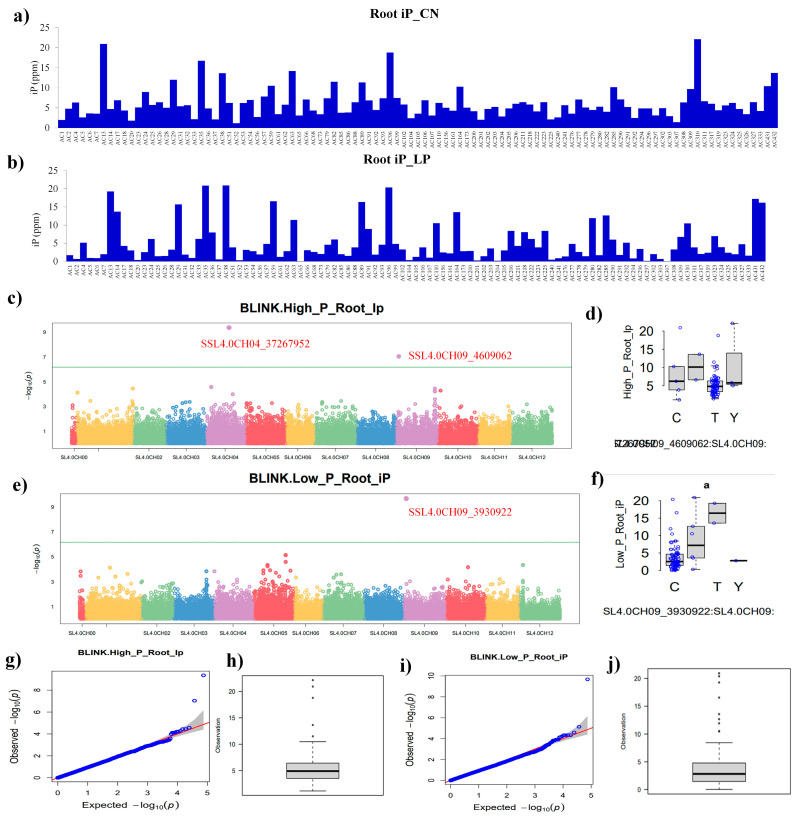
Root iP and GWAS. Root inorganic phosphate content (RiP, ppm/mg fresh weight) of tomato accessions under control (**a**) and low phosphate treatment (**b**), Manhattan plots showing significant SNPs, viz. SSL4.0CH04_37267952 and SSL4.0CH09_4609062, located on chromosome number 4 and 9 in control (**c**), and significant SNPs distribution pattern on chromosome number 9 (**d**), Manhattan plot showing SNP SSL4.0CH09_3930922 on chromosome 9 under low phosphate condition (**e**), and significant SNPs distribution pattern on chromosome number 9 (**f**), the green horizontal line indicates the significance threshold. The quantile–quantile (Q-Q) plots based on observed versus expected −log_10_ (*p*-values) and whisker plots show the behavior of the RiP in control (**g**,**h**) and low phosphate condition (**i**,**j**).

For root inorganic phosphate (RiP), a variation among tomato panel was recorded both in the control and LP conditions (Figure 5a,b). The BLINK model provided two significant SNPs, viz. SSL4.0CH04_37267952 and SSL4.0CH09_4609062, localized on chromosome numbers 4 and 9 above the threshold line in the control conditions (Figure 5c). The distribution pattern of SNPs on chromosomes 4 and 9 under the control conditions is shown in Figure 5d. Furthermore, the Q-Q and whisker plots provided information about the behavior of the RiP trait in an expected-versus-observed manner under the control conditions (Figure 5g,h). The RiP content showed more variation in the tomato panel under the LP condition (Figure 5b). The BLINK model provided a single SNP SSL4.0CH09_3930922 located at chromosome 9 (Figure 5e). The distribution of SNPs on chromosome 9 under the LP conditions is shown in Figure 5f. The Q-Q and whisker plots explained the RiP behavior in an expected-versus-observed manner and the LP condition (Figure 5i,j).

### 3.3. Prediction of Candidate Genes

For the prediction of candidate genes, the genomic region within a range of 70–120 kilobase upstream and downstream of the identified SNP was explored for potential relevance in traits under study, i.e., PB, SiP, and RiP. For plant biomass (PB), significant SNP was detected in the control conditions, while no SNP was detected in LP conditions. The SNP SSL4.0CH10_49261145 for PB under the control conditions when mapped showed the most relevant gene *Solyc10g050370* (Table 1). For SiP under the control conditions, a single SNP, viz. SSL4.0CH08_58433186 located on chromosome 8, was mapped to *Solyc08g074240*. The same trait under the LP conditions showed that one significant SNP, viz. SSL4.0CH08_51271168 on chromosome 8, and two candidate genes, *Solyc08g062490* and *Solyc08g062500,* were mapped. For RiP variation in control P, the GWAS provided two SNPs, i.e., SSL4.0CH04_37267952 and SL4.0CH09_4609062. The SNP SSL4.0CH04_37267952 did not linked to any candidate gene while as SL4.0CH09_4609062 when mapped could be linked with eight candidate genes, viz. *Solyc09g011190*, *Solyc09g011220*, *Solyc09g011240*, *Solyc09g011250*, *Solyc09g011260*, *Solyc09g011320*, *Solyc09g011330*, and *Solyc09g011340*. The higher number of candidate genes for RiP under the control conditions may be attributed to the complexity of the genetic regulation of root P uptake in the tomato panel (Table 1). However, the SNP of RiP under the LP conditions, i.e., SSL4.0CH09_3930922, when mapped was linked to four candidate genes, viz. *Solyc09g010450*, *Solyc09g010460*, *Solyc09g010690*, and *Solyc09g010710*. It was observed that the RiP trait both under the control and LP-provided SNPs localized on chromosome 9, with a cluster of nine genes falling within the range of the 0.6 Mb range of the genomic region of tomato chromosome 9, while SNP localized at chromosome 4 under the control P did not link to any candidate gene. Similarly, for the SiP, two SNPs detected under the control and LP conditions were also localized on chromosome 8 with three genes falling in the genomic region.

### 3.4. Bioinformatic Analysis of Candidate Genes

#### 3.4.1. Plant Biomass (PB)

**Figure 6:**
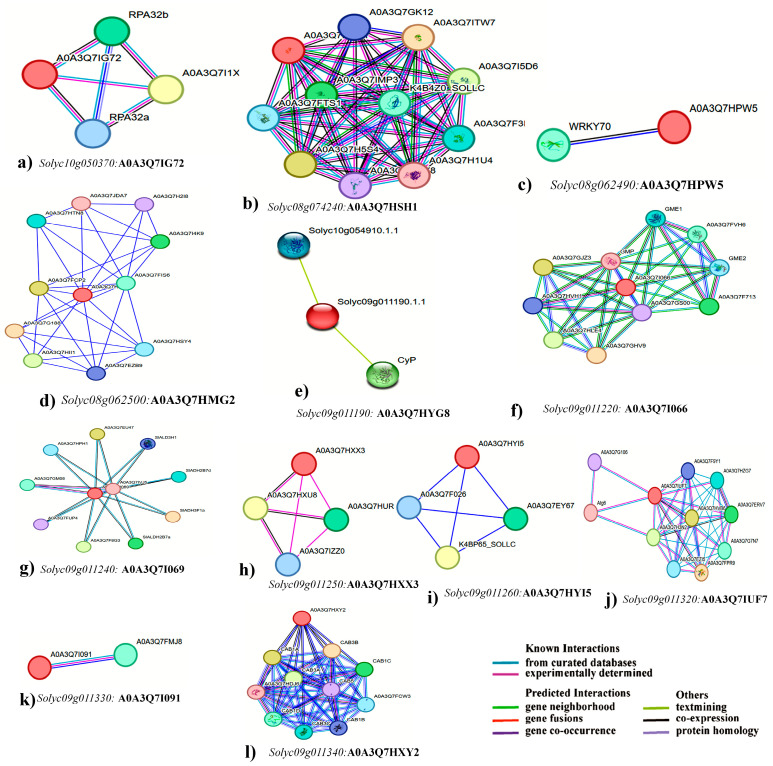
STRING predicted the interaction of proteins. The genes and their protein IDs (shown in bold) show the interaction of proteins of identified candidate genes using STRING database version 12.0 for plant biomass (**a**), shoot inorganic phosphate (SiP, (**b**)) under control, and SiP in low phosphate (**c**,**d**), root inorganic phosphate (RiP) in control (**e**–**l**). The red circle represents the identified candidate gene. The different selection parameters used are shown in colored lines.

The candidate gene *Solyc10g050370* for PB under the control conditions showed that it codes for DNA sequence (CDS) that spanned 1079 base pairs, translating into a 250-amino-acid residue sequence. Its transcript was characterized by four exons, annotated with eight domains, and correlated with 1273 variant alleles, aligning with 24 oligo probes (Table 2). The domain REPLICATION FACTOR A 1, RFA1, possibly orchestrates plant biomass responses to controlled P levels by binding high-efficiency transcription factors [25]. Interactions predicted through the STRING database highlighted three proteins potentially interacting with *Solyc10g050370* in modulating P uptake (Figure 6a).

#### 3.4.2. Shoot Inorganic Phosphate (SiP)

The *Solyc08g074240* gene identified for SiP under the control conditions presented a CDS of 1203 base pairs, encoding 249 amino acids, distributed across six exons. Annotated with 12 domains, associated with 1429 variant alleles, and aligning with 40 oligo probes, its domain small subunit ribosomal protein S6e (RP-S6e, RPS6, Table 2) potentially influences SiP uptake under the controlled P conditions by binding high-efficiency transcription factors. STRING interaction predictions unveiled ten proteins potentially involved in regulating P uptake (Figure 6b). For SiP under the LP conditions, the coding DNA sequence (CDS) of *Solyc08g062490* was 1062 bp, and it encoded 171 amino acid residues. In addition, the transcript has 3 exons, is annotated with 7 domains that are associated with 1226 variant alleles, and maps to 24 oligo probes (Table 2). The domain PTHR31331:SF112 of WRKY TRANSCRIPTION FACTOR 50-RELATED may be involved in responses to low P stress through binding high-P-efficiency transcription factors in plants [23,24]. Predictions of interactions revealed one protein might interact with *SolSolyc08g062490* in the regulation of P uptake under P deficiency using the STRING database (Figure 6c). The coding DNA sequence (CDS) of *Solyc08g062500* for SiP under the LP conditions was 4078 bp, and it encoded 1107 amino acid residues. In addition, the transcript has 3 exons, is annotated with 6 domains that are associated with 1894 variant alleles, and maps to 32 oligo probes (Table 2). Predictions of interactions revealed ten proteins might interact with *Solyc08g062500* in the regulation of P uptake under P deficiency using the STRING database (Figure 6d).

#### 3.4.3. Root Inorganic Phosphate (RiP)

For RiP under the control conditions, the *Solyc09g011190* gene exhibited a CDS of 312 base pairs, encoding 103 amino acids within a single exon. Annotated with three domains, associated with 1285 variant alleles, and mapped to 24 oligo probes, its EXPRESSED PROTEIN domain (Table 2) could contribute to RiP uptake under the controlled P conditions by interacting with high-efficiency transcription factors. Interaction predictions identified two potential proteins interacting with *Solyc09g011190* (Figure 6e). The coding sequence (CDS) of *Solyc09g011220* spans 3849 base pairs, encoding 361 amino acid residues. Furthermore, the transcript comprises 5 exons and is annotated with 8 domains, correlating with 2363 variant alleles and mapped to 19 oligo probes (Table 2). According to predictions from the STRING database depicted in Figure 6f, ten proteins have been identified that potentially interact with *Solyc09g011220*, influencing P uptake regulation. The CDS of *Solyc09g011240* spans 1604 base pairs, directing the synthesis of 317 amino acid residues. Moreover, the transcript is composed of 7 exons, annotated with 15 domains, linked to 1772 variant alleles, and aligned with 37 oligo probes. From the STRING database illustrated in Figure 6g, ten proteins were indicated as potential interactors with *Solyc09g011240*, potentially participating in the regulation of P uptake. In *Solyc09g011250* the CDS was 1256 bp, and it encoded 251 amino acid residues. In addition, the transcript has 4 exons, was annotated with 5 domains that were associated with 2048 variant alleles, and mapped 29 oligo probes. The analysis of interactions indicates that within the regulation of P uptake, three proteins are potentially associated with interacting with *Solyc09g011250*, as highlighted in Figure 6h of the STRING database. Gene *Solyc09g011260* possesses a CDS spanning 2295 base pairs, responsible for encoding 272 amino acid residues. The transcript structure comprises 4 exons and is annotated with 10 domains, correlating with 1471 variant alleles and mapping to 31 oligo probes (Table 2). As indicated by the predictions derived from the STRING database Figure 6i, three proteins have been identified as potential interactors with *Solyc09g011260*, potentially contributing to the regulation of P uptake. *Solyc09g011320* features a coding sequence (CDS) spanning 3086 base pairs, responsible for encoding 711 amino acid residues. The transcript structure encompasses 13 exons and is annotated with 12 domains, linked to 2074 variant alleles, and aligns with 21 oligo probes. According to the predictions from the STRING database displayed in Figure 6j, ten proteins have been identified as potential interactors with *Solyc09g011320*, possibly influencing the regulation of P uptake. *Solyc09g011330* contains a CDS spanning 2313 base pairs, facilitating the encoding of 770 amino acid residues. This transcript structure comprises 2 exons, annotated with 25 domains, correlated with 1500 variant alleles, and correspondingly linked to 26 oligo probes. As per the predictions extracted from the STRING database showcased in Figure 6k, a singular protein has been indicated as potentially interacting with *Solyc09g011330*, contributing to the regulation of P uptake. Lastly, the CDS of *Solyc09g011340* spans 1558 base pairs, encoding 428 amino acid residues. The transcript comprises 7 exons and is annotated with 8 domains, linked to 2102 variant alleles, and mapped to 20 oligo probes (Table 2). According to predictions derived from the STRING database illustrated in Figure 6l, ten proteins have been identified as potential interactors with *Solyc09g011340*, potentially involved in regulating P uptake.

**Figure 7:**
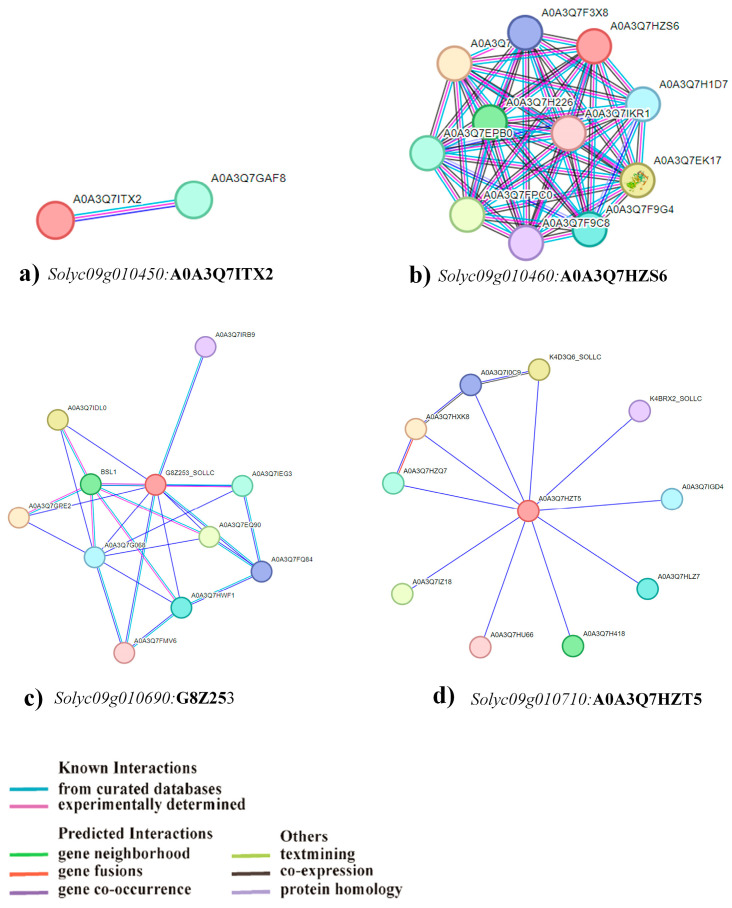
STRING analysis of proteins of candidate genes. The genes and their protein IDs (bold) show the interaction of proteins of the identified candidate gene using STRING database version 12.0 for root inorganic phosphate (RiP) in low phosphate conditions (**a**–**d**). The red circle represents the identified candidate gene. The different selection parameters used are shown in colored lines.

For RiP under the LP conditions, the CDS of *Solyc09g010450* spans 2301 base pairs, encoding 766 amino acid residues. The transcript comprises a single exon and is annotated with 47 domains, linked to 1509 variant alleles, and mapped to 25 oligo probes (Table 2). According to predictions derived from the STRING database, one protein has been identified as a potential interactor with *Solyc09g010450*, potentially involved in regulating P uptake (Figure 7a). The CDS of *Solyc09g010460* spans 4506 base pairs, directing the synthesis of 956 amino acid residues. Moreover, the transcript is composed of 15 exons, annotated with 30 domains, linked to 1973 variant alleles, and aligned with 22 oligo probes (Table 2). The STRING database showed that proteins encoded by this gene could interact with ten proteins that have been indicated as potential interactors with *Solyc09g010460*, potentially participating in the regulation of P uptake (Figure 7b). *Solyc09g010690* contains a CDS spanning 1843 base pairs, facilitating the encoding of 451 amino acid residues. This transcript structure comprises eight exons, annotated with 29 domains, correlated with 1737 variant alleles, and correspondingly linked to 29 oligo probes (Table 2). As per the predictions extracted from the STRING database, ten proteins have been indicated as potentially interacting with *Solyc09g010690*, contributing to the regulation of P uptake (Figure 7c). The *Solyc09g010710* exhibited a CDS of 1350 base pairs, encoding 304 amino acids within a five exon. Annotated with eight domains, associated with 1447 variant alleles, and mapped to 22 oligo probes, its EXPRESSED PROTEIN domain could contribute to RiP uptake under low P conditions by interacting with high-efficiency transcription factors (Table 2). STRING predictions identified ten potential proteins interacting with *Solyc09g010710* (Figure 7d).

### 3.5. In Silico Expression Analysis of Candidate Genes

To further identify the functions of candidate P-efficiency-related genes, the expression levels of a few candidate genes are based on low P-induced transcriptome analysis in tomato [22]. For instance, *Solyc08g062490* was negatively induced by P deficiency in the root under low P conditions, while it was positively induced in the shoot (Figure S4). Gene *Solyc08g062500* was positively induced in the root while no expression was observed in the shoot. *Solyc09g010710* was negatively induced in both root and shoot, while *Solyc09g010690* was expressed differentially in root and shoot. *Solyc09g010460* was negatively induced in the root while as no expression was observed in shoot. *Solyc09g010450* was negatively induced in both root and shoot under low P conditions (Figure S4).

## 4. Discussions

Since its domestication, tomato has undergone extensive changes leading to the development of diverse morphotypes for plant architecture [26]. Plant breeders have long focused on traits with the potential to increase yields while decreasing inputs biology [27]. Tomatoes hold a vital place as a nutrient-rich and fibrous component in the human diet, while also serving as an exemplary model for delving into biological research [28]. The increased demand for tomatoes has led to a large portion of agricultural land being put into tomato cultivation. To increase the yield, unchecked quantities of fertilizers are being used by farmers. Fertilizer such as phosphate is a non-renewable mineral resource that is extensively used in tomato cultivation. Identification of novel candidate genes that may be putatively associated with root and shoot P content and morphometry is of vital importance in tomato.

The shoot height in tomato seedlings is an important factor for plant growth, as it is an indicator of the plant’s overall development and health [28]. In our studies, shoot height was found to be enhanced in maximum tomato accessions showing their capability to uptake phosphate as a key macro-element for plant growth and development. In earlier studies, it was found that phosphate fertilization enhanced the plant height in tomato [29]. Similar studies regarding increased phosphate fertilization showed a significant increase in plant height [30]. Low P availability inhibits crop productivity in many low-input agricultural systems, particularly in places where calcareous and alkaline soils predominate. Improving P fertilizer use efficiency is thus critical for developing plants with higher phosphorous uptake and utilization [31]. In our studies, it was found that PRL was increased in seedling stages at the control P conditions similar to previous studies conducted on tomato, and PRL was found to be enhanced in tomato seedlings on P availability [32]. The obtained results coincide with numerous previous studies as it has been reported that inbred lines that exhibit tolerance to low phosphorus (LP) conditions typically demonstrate extended root length, increased root volume, expanded leaf area, and a higher density of root hairs compared to their counterparts with sensitivity to P deficiency [33]. Our studies showed that plant biomass was significantly increased in maximum accessions showing their enhanced phosphate uptake mechanism, which contributed to the overall plant growth.

Similar results were obtained in studies under the P application that showed enhanced plant biomass in tomato [34]. Other studies also revealed that, on applying synergistic application of phosphate solubilizing bacteria and different phosphate amendments, a significant increase was observed in plant biomass and P uptake [35]. The concentration of phosphorus (P) in plant tissues and its impact on growth and nutrient acquisition can be influenced by intricate interplays among climate, soil characteristics, and agricultural management practices [36]. Within the intricate web of interactions involving soil and agricultural management, soil pH emerges as a crucial factor influencing the conversion of organic phosphorus (P) into soluble P [37]. Comprehending the intricate dynamics of nutrient synergies and potential antagonisms within the rhizosphere is essential in addressing worldwide challenges associated with climate change and population growth. This understanding is pivotal in our efforts to mitigate the environmental footprint of agricultural practices [38]. Ensuring optimal uptake of nutrients by plants stands as a pivotal factor in guaranteeing both sufficient crop yields and the quality of the harvest [39].

Similar to our studies, a GWAS was carried out for shoot dry weight-related traits using 3.66 million high-quality single nucleotide polymorphisms (SNPs). They identified the *Gh_D11G2219* gene, which plays a significant role in the regulation of tolerance to P deficiency stress in cotton [9]. Moreover, through an analysis encompassing 277 accessions of *A. thaliana*, association studies and complementary genomic techniques were employed to scrutinize root growth rates. This comprehensive approach resulted in the identification of three genes responsible for modulating the variability in root growth under conditions of phosphorus deficiency, either independently or in conjunction with other nutrient scarcities, utilizing GWAS [40]. The recent GWAS conducted in *Brassica napus* had successfully pinpointed 12 genes linked to root growth and development under low phosphorus (LP) stress. Out of the identified set of twelve genes, six (*BnaA04g23490D*, *BnaA09g08440D*, *BnaA09g04320D*, *BnaA09g04350D*, *BnaA09g04930D*, *BnaA09g09290D*) showcased distinct patterns of expression, signifying their potential as prime candidate genes linked to the desired traits [41]. Similar studies regarding the GWAS on plant biomass were conducted in soybeans, deciphering in total 10 loci encompassing 47 putative candidate genes. Within these genetic loci, seven instances of domestication sweep and six improvement sweeps have been identified. Notably, *Glyma.05G047900*, encoding a purple acid phosphatase, has emerged as a robust candidate gene for enhancing biomass, offering promising prospects for the future of soybean breeding efforts [42]. Previous research on mungbean identified a total of 71 protein-coding genes. Among these, 13 genes were recognized as potential candidates governing phosphate use efficiency (PUE) through the regulation of nutrient uptake and root architectural development pathways. Notably, from this subset of thirteen genes, three stand out as potential candidates—*VRADI11G08340*, *VRADI01G05520*, and *VRADI04G10750*—for enhancing low phosphorus tolerance in mung bean [43].

In a separate investigation conducted on *Brassica juncea*, the analysis of genomic regions surrounding significantly associated SNPs enabled the prediction of 15 potential candidates. This set encompassed various gene families, notably those encoding acid phosphatases such as *PAP10* (purple acid phosphatase 10), *PAP16*, *PNP* (polynucleotide phosphorylase), and *AT5G51260* (HAD superfamily gene, subfamily IIIB acid phosphatase) [44]. The above-identified eight genes, i.e., *Solyc09g011190*, *Solyc09g011220*, *Solyc09g011240*, *Solyc09g011250*, *Solyc09g011260*, *Solyc09g011320*, *Solyc09g011330* and *Solyc09g011340*, in RiP using the GWAS might be playing role in phosphate homeostasis.

## 5. Conclusions

The present study has provided valuable insights into the phenotypic variations of 103 tomato accessions when subjected to varying phosphate conditions, specifically focusing on parameters like shoot height, primary root length, plant biomass, shoot inorganic phosphate, and root inorganic phosphate. Through a comprehensive analysis of these parameters, we were able to identify those tomato accessions that exhibited distinct levels of phosphate uptake, shedding light on the genetic diversity within this plant species. Through the implementation of the GWAS and the BLINK model, we have successfully identified six SNPs (1 in PB, 1 in SiP, and 2 in RiP under control P, and 1 in SiP, and 1 in RiP under low P) associated with sixteen candidate genes (1 in PB, 3 in SiP, and 12 in RiP) that may play pivotal roles in the intricate network governing phosphate homeostasis. As to future studies, further research can delve deeper into the functional validation of these candidate genes and explore their potential applications in breeding programs. This study serves as a foundation for ongoing efforts to enhance crop productivity and adaptability in the face of changing environmental conditions, ensuring a more sustainable and food-secure future.

## Figures and Tables

**Table 1:** Mapping of SNPs for candidate genes. List of single nucleotide polymorphisms (SNPs) provided by Bayesian Linear Regression for Mixed Model Association (BLINK) in tomato accessions subjected to normal (control P, CN) and low phosphate (LP) conditions for two weeks. The BLINK model analyzed plant biomass (PB, g), shoot inorganic phosphate (SiP, ppm/mg fresh weight), and root inorganic phosphate (RiP, ppm/mg fresh weight). The location of the SNP number, location on the chromosome, its position, p-value, and minimum allele frequency (MAF) are provided.

	Trait	SNP	Chromosome	Position	*p*-Value	MAF	Genes
PB	CN	SSL4.0CH10_49261145	10	49261145	2.67 × 10^−12^	0.333333	*Solyc10g050370*
	LP	No significant SNP	-	-	-	-	-
SiP	CN	SSL4.0CH08_58433186	8	58433186	4.96 × 10^−11^	0.059524	*Solyc08g074240*
	LP	SSL4.0CH08_51271168	8	51271168	1.99 × 10^−9^	0.083333	*Solyc08g062490* *Solyc08g062500*
RiP	CN	SSL4.0CH04_37267952 SL4.0CH09_4609062	4 9	37267952 4609062	4.41 × 10^−10^ 9.20 × 10^−8^	0.255952 0.089286	- *Solyc09g011190* *Solyc09g011220* *Solyc09g011240* *Solyc09g011250* *Solyc09g011260* *Solyc09g011320* *Solyc09g011330* *Solyc09g011340*
	LP	SSL4.0CH09_3930922	9	3930922	2.10 × 10^−10^	0.065476	*Solyc09g010450* *Solyc09g010460* *Solyc09g010690* *Solyc09g010710*

**Table 2:** Relevant genes associated with phenotypic traits were analyzed. Each single nucleotide polymorphism (SNP) was mapped within a 70–120 kb region for traits, viz. plant biomass (PB), shoot inorganic phosphate (SiP), root inorganic phosphate (RiP) measured under normal (control, CN) and low phosphate (LP) conditions using BLINK model of GWAS. Relevant genes with ID are provided along with their details at https://phytozome-next.jgi.doe.gov/ (accessed on 3 October 2023). Candidate genes were also supported with homologous genes in *Arabidopsis thaliana*.

Trait	Treatment	Gene ID	Phytozome	Homologous Genes in *Arabidopsis*
PB	CN	*Solyc10g050370*	Replication factor A1, RFA1	*AT2G05642,* Nucleic acid-binding, OB-fold protein
SiP	CN	*Solyc08g074240*	Small subunit ribosomal protein S6e	*AT4G31700*.1: ribosomal protein S6
	LP	*Solyc08g062490*	WRKY transcription factor 50	*At5g26170*: WRKY DNA-BINDING
		*Solyc08g062500*	N-terminal C2 in EEIG1 and EHBP1 proteins (NT-C2)	*At5g20610*: N-terminal C2 in EEIG1 and EHBP1 proteins (NT-C2)
RiP	CN	*Solyc09g011190*	Expressed protein	*AT5G02090*.1: PTHR34670:SF3
		*Solyc09g011220*	Mannose-1-phosphate guanylyl transferase 3-related	*AT2G39770*.1: cytokinesis defective 1 embryo defective 101 GDP-mannose pyro phosphorylase 1
		*Solyc09g011240*	Aldo-keto reductase family4	*AT2G37790*.1:3-beta-(or20-alpha)-hydroxysteroid dehydrogenase
		*Solyc09g011250*	Glycoprotein family protein	*AT3G55605*.1: K15414—complement component 1 Q subcomponent-binding protein
		*Solyc09g011260*	Cysteine/histidine-richC1 domain family protein-related	*AT2G37810*.1: PTHR13871//PTHR13871:SF30—thioredoxin
		*Solyc09g011320*	Serine/threonine-protein kinase ULK/ATG1	*AT2G37840*.1: K08269-serine/threonine-protein kinase ULK/ATG1
		*Solyc09g011330*	PAN domain (PAN_1)//Protein kinase domain (Pkinase)	*AT2G19130*.1: S-locus lectin protein kinase family protein
		*Solyc09g011340*	Domain of unknown function (DUF3411)	*AT2G37860*.3: LOWER CELL DENSITY 1, Protein of unknown function (DUF3411)
	LP	*Solyc09g010450*	Pentatricopeptide repeat Tetratricopeptide-like helical domain	*At3g53700*: maternal effect embryo arrest 40/Pentatricopeptide repeat (PPR) superfamily protein
		*Solyc09g010460*	translation initiation factor 3 subunit A (EIF3A)	*At3g53700*: maternal effect embryo arrest 40/Pentatricopeptide repeat (PPR) superfamily protein
		*Solyc09g010690*	E3 Ubiquitin-protein ligase XBAT31-related	*At2g28840*: Putative E3 Ub protein ligase
		*Solyc09g010710*	Unknown Protein	*At3g09430*: peptide transporter family protein

## Data Availability

All the data used for the preparation of this manuscript were provided in the main and Supplementary Files. The genotype-by-sequencing (GBS) data can be provided upon request.

## Supplementary Materials

The following supporting information can be downloaded at: https://www.mdpi.com/article/10.3390/plants13030457/s1, Figure S1: Trait distribution [shoot height (a), plant biomass (b), and primary root length (c)] in tomato panel subjected to the control and low phosphate conditions for two weeks; Figure S2: Distribution pattern of shoot (a) and root (b) inorganic phosphate in tomato accessions subjected to the control and low phosphate treatment for two weeks; Figure S3: Segregation of tomato accessions into tolerant and sensitive accessions based on shoot Pi, root Pi, and root/shoot ratio under the control (P+) and low phosphate (P−) conditions; Figure S4: Relative gene expression of candidate genes in root and shoot tissue in low phosphate condition compared to the control conditions (taken from transcriptomic data of tomato published by Satheesh et al. [22]); Table S1: Distribution of shoot height (cm), plant dry biomass (g), and primary root length (cm) of tomato panel subjected to the control and low phosphate treatment for two weeks; Table S2: Prediction of interacting protein using STRING database version 12.0 for plant biomass, shoot inorganic phosphate (SiP), and root inorganic phosphate (RiP) under the control P condition; Table S3: Prediction of interacting protein using STRING database version 12.0 for shoot inorganic phosphate (SiP), and root inorganic phosphate (RiP) under low P condition.
