# Supplementary material for: Genome-Wide Association Study (GWAS) for Identifying SNPs and Genes Related to Phosphate-Induced Phenotypic Traits in Tomato (Solanum lycopersicum L.)
Source: Plants (Basel). 2024 Feb 5;13(3):457. doi: 10.3390/plants13030457 (PMC10857258; doi:10.3390/plants13030457)
Supplement: Supplementary file 1 [file plants-13-00457-s001.zip › plants-2809770-supplementary.pdf]

Supplementary file

Genome-Wide Association Study (GWAS) for Identifying SNPs and Genes Related to Phosphate-Induced Phenotypic Traits in Tomato (*Solanum lycopersicum* L.)

Haroon Rashid Hakla [1,2], Shubham Sharma [1], Mohammad Urfan [1], Rushil Mandlik [3,4], Surbhi Kumawat [3,5], Prakriti Rajput [1], Bhubneshwari Khajuria [1], Rehana Chowdhary [1], Rupesh Deshmukh [3,4], Rajib Roychowdhury [6,*] and Sikander Pal [1,*]

1 Plant Physiology Laboratory, Department of Botany, University of Jammu, Jammu 180006, India; haroonhakla@rediffmail.com (H.R.H.); shubhamsharma199425@gmail.com (S.S.); urfanbutt1992@gmail.com (M.U.); prakritirajput2512@gmail.com (P.R.); bhubneshwarikhajuria@gmail.com (B.K.); rehanachowdhary2937@gmail.com (R.C.)
2 Central Integrated Pest Management Centre (CIPMC), Srinagar 190008, India
3 ICAR-National Bureau of Plant Genetic Resources, New Delhi 110012, India; rushilmandlik91@gmail.com (R.M.); surbhikumawat002@gmail.com (S.K.); rupesh0deshmukh@gmail.com (R.D.)
4 Department of Biotechnology, Central University of Haryana, Mahendergarh 123031, India
5 Department of Ecology and Evolutionary Biology, University of Kansas, Lawrence, KS 66045, USA
6 Department of Plant Pathology and Weed Research, Institute of Plant Protection, Agricultural Research Organization (ARO)—Volcani Center, Rishon LeZion 7505101, Israel
* Correspondence: rajibroychowdhury86@yahoo.com (R.R.); sikanderpal@jammuuniversity.ac.in (S.P.)

Supplementary Table

Table S1. Distribution of shoot height (cm), plant dry biomass (g), and primary root length (cm) of tomato panel subjected to control and low phosphate treatment for two weeks.

Accession code	Shoot height (cm)		Plant dry biomass (g)		Primary root length (cm)	
	Control	Low phosphate	Control	Low phosphate	Control	Low phosphate
AC1	4.68	3.5	0.16	0.1	8.52	3.62
AC102	6.1	6.44	0.25	0.26	11.66	12.3
AC104	5.4	4.68	0.21	0.21	12.38	9.42
AC105	5.56	4.72	0.25	0.18	11.22	9.3
AC106	5.48	5.52	0.21	0.18	11.4	8.48
AC107	5.22	4.56	0.18	0.16	9.58	7.76
AC110	4.82	5.98	0.21	0.23	7.66	10.6
AC13	4.86	6.12	0.12	0.17	10.4	8.94
AC14	4.46	5.52	0.15	0.18	9.74	10.68
AC156	6.58	6.88	0.31	0.33	12.8	11.4
AC161	6.04	5.54	0.21	0.19	12.34	11.8
AC164	4.36	5.8	0.1	0.13	5.9	7.46
AC17	4.14	3.7	0.1	0.05	8.18	3.74
AC173	5.08	4.68	0.18	0.19	7.7	11.9
AC18	5.38	6.24	0.26	0.18	11.38	8.38
AC2	5.04	4.12	0.19	0.07	12.26	5.28
AC20	6.36	4.58	0.23	0.11	10.48	5.54
AC200	5.46	4.9	0.24	0.21	12.14	8.24
AC201	5.32	4.64	0.31	0.26	10.86	8.12
AC202	5.98	4.74	0.3	0.25	8.98	7.22
AC203	4.94	4.14	0.17	0.14	8.24	6.88
AC204	5.8	4.84	0.23	0.22	10.92	9.54

AC205	5.2	5.82	0.25	0.23	8.6	10.64
AC206	5.2	5.64	0.21	0.23	9.22	10.64
AC211	6.12	5.54	0.26	0.23	11.92	10.42
AC218	6.38	6.54	0.28	0.35	14.02	13.54
AC222	4.88	5.54	0.21	0.23	9.46	11.66
AC223	3.48	2.4	0.14	0.11	6.66	3.04
AC225	3.6	5.72	0.15	0.19	3.46	11.22
AC23	4.96	4.06	0.15	0.12	9.24	4.14
AC24	4.6	4	0.14	0.09	8.2	6.22
AC240	3.94	3.1	0.15	0.13	4.96	3.44
AC241	4.42	3.44	0.2	0.15	4.96	2.72
AC25	5.46	4.06	0.15	0.13	8.44	5.72
AC26	6.38	5.44	0.24	0.15	10.38	7.44
AC276	5.16	4.38	0.18	0.18	11.88	6.02
AC277	5.62	5.3	0.15	0.14	9.58	6.28
AC278	5.46	4.8	0.16	0.15	10.38	6.12
AC279	4.52	4.2	0.18	0.16	9.76	6.02
AC28	5.42	4.72	0.21	0.14	11.08	6.72
AC280	5.24	5.9	0.19	0.21	10.5	7.74
AC282	6.02	5.14	0.2	0.15	10.88	7.36
AC285	4.52	5.12	0.14	0.18	9.3	7.42
AC29	5.52	5.82	0.19	0.22	10.38	9.12
AC290	5.48	4.78	0.18	0.16	9.46	7.96
AC291	5.54	4.76	0.19	0.17	9.86	7.88
AC292	3.46	3.76	0.15	0.13	4.92	6.18
AC294	5.54	4.54	0.25	0.21	10.48	7.86
AC296	5.28	4.3	0.15	0.11	10.52	2.68
AC297	4.28	3.86	0.12	0.11	7.02	5.94
AC302	5.16	4.38	0.2	0.15	8.38	6.26
AC303	5.78	5.1	0.22	0.17	10	6.24
AC307	5.58	4.98	0.21	0.19	9.24	7.78
AC308	4.32	4.06	0.18	0.12	6.66	4.96
AC309	5.3	3.76	0.25	0.21	12.16	8.14
AC31	5.02	3.88	0.13	0.11	9.06	6.04
AC310	6.54	6.94	0.3	0.35	10.6	10.28
AC311	6.06	3.96	0.15	0.11	10.4	5.32
AC317	5.98	4.96	0.15	0.12	8.3	6.84
AC319	4.08	4.26	0.16	0.17	6.62	7.22
AC32	4.9	4.12	0.19	0.13	8.52	5.54
AC323	5.28	3.52	0.15	0.09	10.52	4.78
AC324	4.5	4.96	0.15	0.15	8.26	7.1
AC325	4.8	4.12	0.25	0.22	10.72	6.82
AC326	6.52	5.4	0.25	0.24	9.8	10.68
AC327	5.18	5.04	0.2	0.18	10.88	3.62
AC33	3.92	4.8	0.13	0.16	4.3	6.06
AC333	5.3	4.72	0.17	0.12	9.78	8.66
AC35	5.12	5.98	0.21	0.25	10.24	11.68
AC36	4.86	5.78	0.18	0.21	9.28	9.18
AC37	4.64	4.08	0.17	0.15	5.96	4.8
AC38	6.16	4.24	0.25	0.15	9.46	5.18
AC4	4.74	3.84	0.18	0.12	8.08	5.7

AC431	5.68	6.08	0.24	0.28	10	10.1
AC432	6.04	6.68	0.26	0.29	12.68	10.24
AC5	4.6	5.58	0.15	0.1	4.86	7.82
AC51	5.6	4.52	0.27	0.19	11.22	6.92
AC52	4.56	3.52	0.11	0.08	8.42	4.52
AC53	6.44	5.48	0.24	0.23	11.1	8.38
AC54	5.82	4.36	0.23	0.21	13.54	7.46
AC56	5.5	4.64	0.13	0.12	9.02	7.38
AC57	5.26	4.46	0.19	0.15	10.72	7.04
AC59	4.98	5.16	0.12	0.16	8.58	6.82
AC6	5.9	5.1	0.21	0.16	11.7	6.24
AC61	5.56	4.84	0.21	0.2	11.52	6.62
AC62	5	4.3	0.12	0.11	9.02	6.62
AC63	4.04	4.74	0.11	0.13	3.88	7.5
AC65	6.22	5.3	0.22	0.2	10.74	8.1
AC66	5.96	5.84	0.25	0.23	13	11.42
AC68	6	4.52	0.24	0.19	9.16	4.96
AC7	3.94	3.28	0.13	0.1	6.5	4.54
AC73	5.72	5.24	0.28	0.26	12.72	9.6
AC79	5.42	4.58	0.25	0.21	12.18	7.52
AC82	5.86	3.62	0.24	0.11	11.48	6.76
AC85	5.36	4.86	0.16	0.17	11.4	9.72
AC86	6.88	6.3	0.28	0.25	14.34	13.2
AC88	5.6	5.94	0.21	0.22	9.56	10.3
AC89	5.6	5.94	0.17	0.2	9.36	10.6
AC91	4.98	5.18	0.23	0.25	9.12	10.18
AC92	5.28	4.72	0.15	0.14	9.14	7.58
AC93	4.5	4.1	0.17	0.15	7.1	5.46
AC96	6.34	3.98	0.25	0.14	11.64	5.7
AC99	6	6.54	0.22	0.25	11.9	12.12

Table S2. Prediction of interacting protein using STRING database version 12.0 for plant biomass, shoot inorganic phosphate (SiP), and root inorganic phosphate (RiP) under control P condition.

Trait	Candidate Gene	Candidate Protein	Predicted Interacting Protein
**Plant Biomass**	*Solyc10g050370*	A0A3Q7IG72	A0A3Q7I1X9, RPA32a, and RPA32b
**SiP**	*Solyc08g074240*	A0A3Q7HSH1	A0A3Q7GK12, A0A3Q7ITW7, A0A3Q7I5D6, K4B4Z0_SOLLC, A0A3Q7F3F2, A0A3Q7H1U4, A0A3Q7GRY8, A0A3Q7H5S4, A0A3Q7FTS1, and A0A3Q7IMP3
**RiP**	*Solyc09g011190*	A0A3Q7HYG8	A0A3Q7JCM7, and P21568
	Solyc09g011220	A0A3Q7I066	GME2, GME1, A0A3Q7F713, A0A3Q7IKE4, A0A3Q7GHV9, A0A3Q7GS00, A0A3Q7HLE4, A0A3Q7HVH1, A0A3Q7GJZ3, and A0A3Q7FVH6
	Solyc09g011240	A0A3Q7I069	A0A3Q7EU47, SlALD3H1, SlADH2B7d, SlADH3F1a, SlALDH2B7a, A0A3Q7F8G3, A0A3Q7FUP4, A0A3Q7GM08, A0A3Q7HPH1, and A0A3Q7IVJ5
	Solyc09g011250	A0A3Q7HXX3	A0A3Q7HUR3, A0A3Q7IZZ0, and A0A3Q7HXU8
	Solyc09g011260	A0A3Q7HYI5	A0A3Q7EY67, K4BP65_SOLLC, and A0A3Q7F026
	Solyc09g011320	A0A3Q7IUF7	A0A3Q7F9Y1, A0A3Q7HZG7, A0A3Q7ERV7, A0A3Q7G7N7, A0A3Q7FPR9, A0A3Q7EZI5, A0A3Q7HVB6, A0A3Q7H3N2, A0A3Q7G106, and Atg6

	Solyc09g011340	A0A3Q7HXY2	A0A3Q7HXY2, CAB3B, CAB1C, A0A3Q7FCW3, CAB1B, CAB3C, CAB1D, CAB5, A0A3Q7HDJ6, CAB3A, and CAB1A

Table S3. Prediction of interacting protein using STRING database version 12.0 for shoot inorganic phosphate (SiP), and root inorganic phosphate (RiP) under low P condition.

Trait	Candidate Gene	Candidate Protein	Predicted Interacting Protein
**SiP**	*Solyc08g062490*	A0A3Q7HPW5	WRKY70
	Solyc08g062500	A0A3Q7HMG2	A0A3Q7JDA7, A0A3Q7H2I8, A0A3Q7I4K9, A0A3Q7FIS6, A0A3Q7HSY4, A0A3Q7EZB9, A0A3Q7HII1, A0A3Q7G188, A0A3Q7FCP2, A0A3Q7HTN8, and A0A3Q7JDA7
**RiP**	*Solyc09g010450*	A0A3Q7ITX2	A0A3Q7GAF8
	Solyc09g010460	A0A3Q7HZS6	A0A3Q7F3X8, A0A3Q7H1D7, A0A3Q7EK17, A0A3Q7F9G4, A0A3Q7F9C8, A0A3Q7FPC0, A0A3Q7EPB0, A0A3Q7IKR1, A0A3Q7H226, and A0A3Q7G5L4
	Solyc09g010690	G8Z253	A0A3Q7IRB9, A0A3Q7IEG3, A0A3Q7EQ90, A0A3Q7FQ84, A0A3Q7HWF1, A0A3Q7FMV6, A0A3Q7G068, A0A3Q7GPE2, BSL1, and A0A3Q7IDL0
	Solyc09g010710	A0A3Q7HZT5	K4D3Q6_SOLLC, K4BRX2_SOLLC, A0A3Q7IGD4, A0A3Q7HLZ7, A0A3Q7H418, A0A3Q7HU66, A0A3Q7IZ18, A0A3Q7HZQ7, A0A3Q7HXK8, and A0A3Q7I0C9

Supplementary Figures

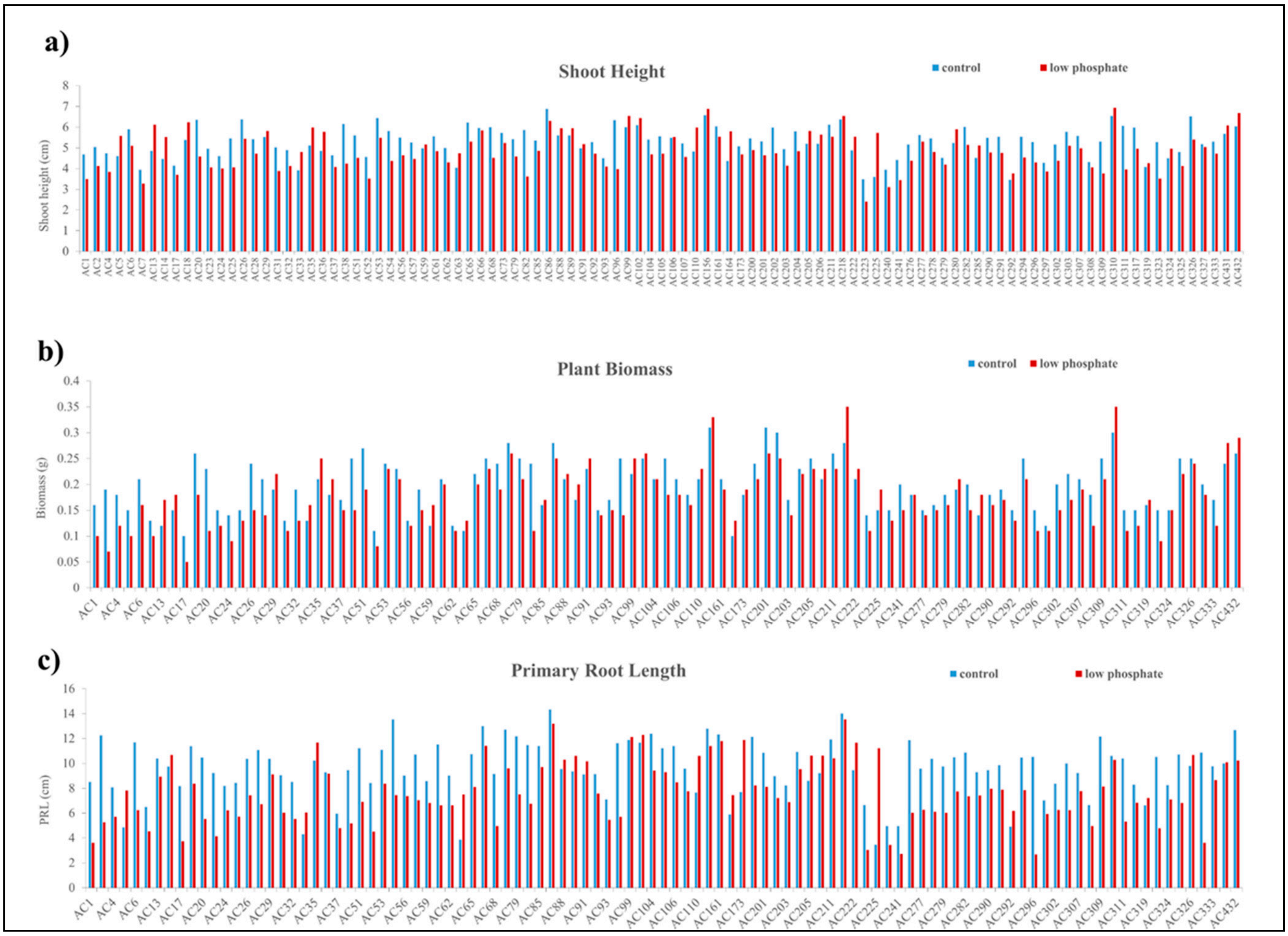

Figure S1. Trait distribution [shoot height (a), plant biomass (b), and primary root length (c)] in tomato panel subjected to control and low phosphate conditions for two weeks.

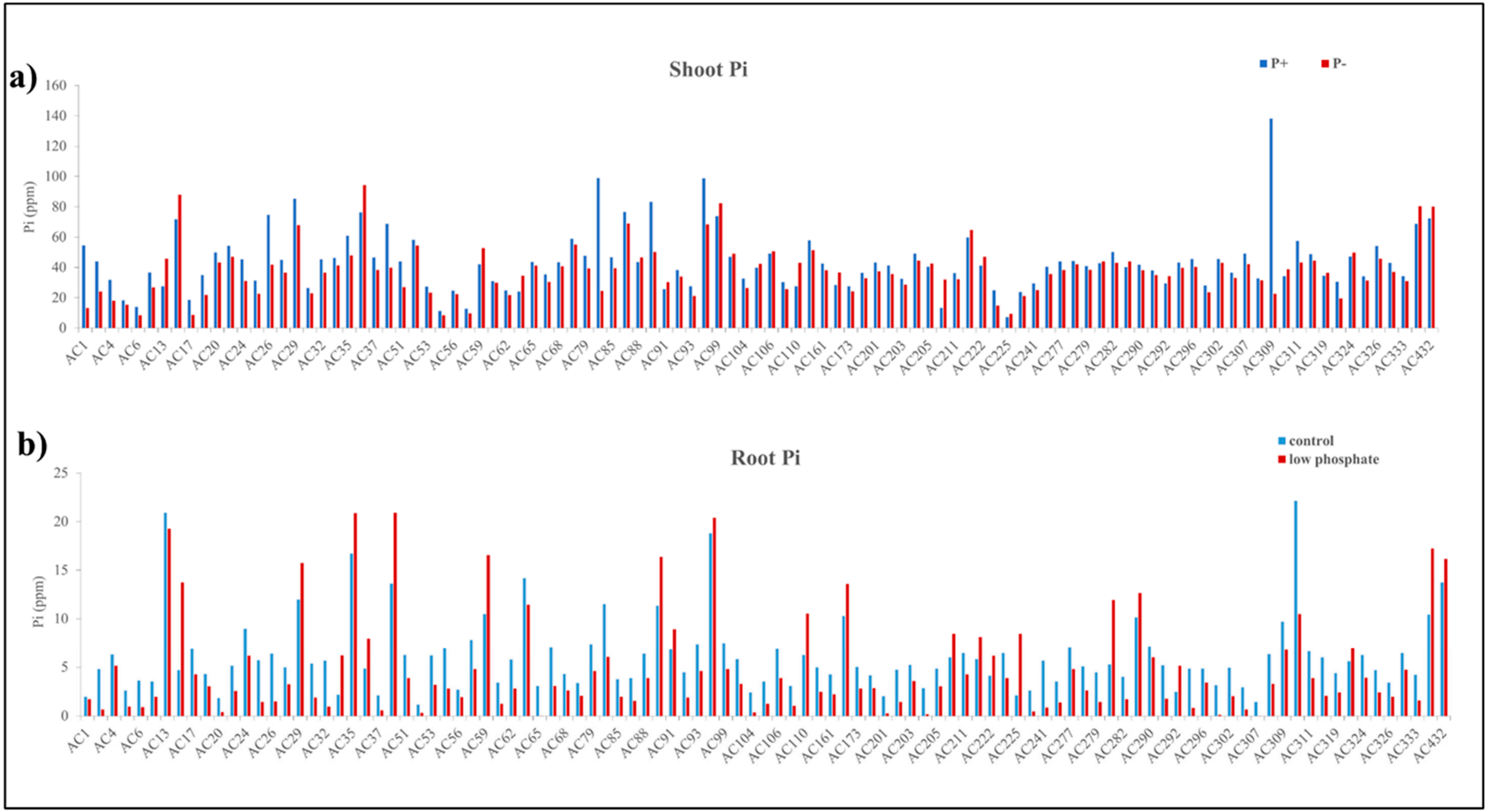

Figure S2. Distribution pattern of shoot (a) and root (b) inorganic phosphate in tomato accessions subjected to control and low phosphate treatment for two weeks.

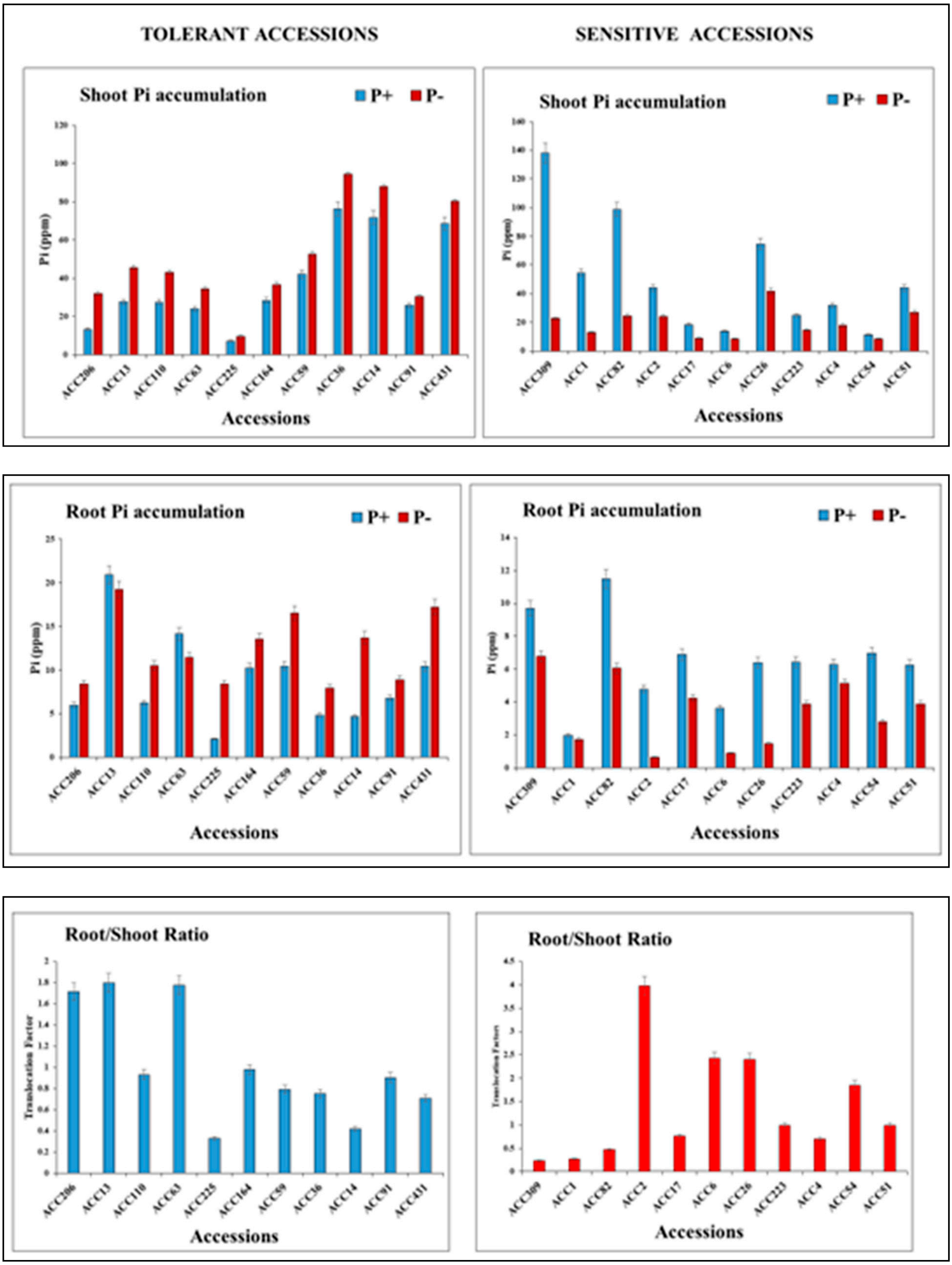

Figure S3. Segregation of tomato accessions into tolerant and sensitive accessions based on shoot Pi, root Pi, and root/shoot ratio under control (P+) and low phosphate (P-) conditions.

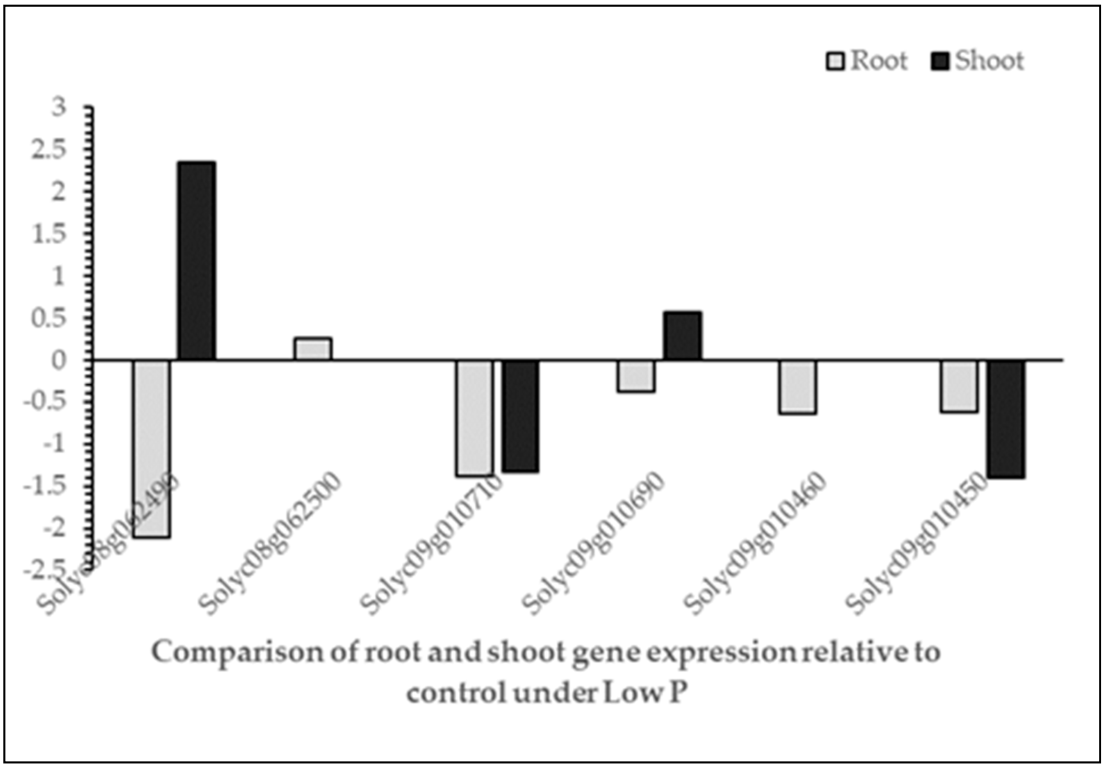

Figure S4. Relative gene expression of candidate genes in root and shoot tissue in low phosphate condition compared to control (taken from transcriptomic data of tomato published by Satheesh et al. [22]).
